# Plant-Based Diets Improve Maternal–Fetal Outcomes in CKD Pregnancies

**DOI:** 10.3390/nu14194203

**Published:** 2022-10-09

**Authors:** Rossella Attini, Filomena Leone, Antoine Chatrenet, Elisa Longhitano, Viola Casula, Alice Tomasi Cont, Gaia Zaccaria, Eleonora Dalmasso, Ana Maria Manzione, Bianca Masturzo, Massimo Torreggiani, Alberto Revelli, Gianfranca Cabiddu, Giorgina Barbara Piccoli

**Affiliations:** 1 Department of Obstetrics and Gynecology SC2U, Città della Salute e della Scienza, Sant’Anna Hospital, 10126 Turin, Italy; 2 Néphrologie et Dialyse, Centre Hospitalier Le Mans, 72037 Le Mans, France; 3 Laboratory “Movement, Interactions, Performance” (EA 4334), Le Mans University, 72000 Le Mans, France; 4 Department of Clinical and Experimental Medicine, Unit of Nephrology and Dialysis, A.O.U. “G. Martino”, University of Messina, 98124 Messina, Italy; 5 Nephrology, Dialysis and Renal Transplantation Division, Department of Medical Sciences, “Città della Salute e della Scienza” Hospital, University of Turin, 10100 Turin, Italy; 6 Nephrology, Department of Medical Science and Public Health, San Michele Hospital, G. Brotzu, University of Cagliari, 09047 Cagliari, Italy

**Keywords:** pregnancy complications, preterm delivery, small for gestational age, preeclampsia, chronic kidney disease, plant-based diets

## Abstract

Reducing protein intake in patients with chronic kidney disease (CKD) limits glomerular stress induced by hyperfiltration and can prevent the progression of kidney disease; data in pregnancy are limited. The aim of this study is to analyze the results obtained in CKD patients who followed a plant-based moderately protein-restricted diet during pregnancy in comparison with a propensity-score-matched cohort of CKD pregnancies on unrestricted diets. A total of 52 CKD pregnancies followed up with a protein-restricted plant-based diet (Torino, Italy) were matched with a propensity score based on kidney function and proteinuria with CKD pregnancies with unrestricted protein intake (Cagliari Italy). Outcomes included preterm (<37 weeks) and very preterm (<34 weeks) delivery and giving birth to a small-for-gestational-age baby. The median age in our cohort was 34 years, 63.46% of women were primiparous, and the median body mass index (BMI) was 23.15 kg/m^2^ with 13.46% of obese subjects. No statistical differences were found between women on a plant-based diet and women who were not in terms of age, parity, BMI, obesity, CKD stage, timing of referral, or cause of CKD. No differences were found between the two groups regarding the week of delivery. However, the combined negative outcome (birth before 37 completed gestational weeks or birth-weight centile <10) occurred less frequently in women following the diet than in women in the control group (61.54% versus 80.77%; *p* = 0.03). The lower risk was confirmed in a multivariable analysis adjusted for renal function and proteinuria (OR: 0.260 [Q1:0.093–Q3:0.724]; *p* = 0.010), in which the increase in proteinuria from the first to the last check-up before delivery was lower in patients on plant-based diets (median from 0.80 to 1.87 g/24 h; p: ns) than in controls (0.63 to 2.39 g/24 h *p* < 0.0001). Plant-based, moderately protein-restricted diets in pregnancy in patients with CKD are associated with a lower risk of preterm delivery and small-for-gestational-age babies; the effect may be mediated by better stabilization of proteinuria.

## 1. Introduction

Chronic kidney disease (CKD) is defined as a reduction in the kidney function corresponding to an estimated glomerular filtration rate (eGFR) below 60 mL/min or any alteration of renal morphology, including malformations, kidney stones, or kidney scars, or a change in the composition of the urine (such as proteinuria or hematuria) or the blood (such as renal tubular acidosis), independently from kidney function, lasting for at least 3 months [1]. It is estimated that it is present in about 3% of all pregnancies, with about 1:750 occurring in women in an advanced CKD stage (eGFR < 60 mL min, corresponding to CKD stages 3-4-5) [2,3].

Independently from kidney function, the presence of kidney damage, or a reduction in kidney tissue, it is now known to be associated with an increased incidence of adverse pregnancy outcomes, including preterm delivery, preeclampsia, the hypertensive disorders of pregnancy, and giving birth to a small-for-gestational-age (SGA) baby [4,5,6]. This is also true of those who have a healthy single kidney (as is the case of kidney donors), and is observed, among others, in patients with kidney stones, and in cases with a previous episode of acute kidney injury (AKI), even after complete normalization of renal function [7,8,9,10]. The risk of adverse pregnancy outcomes is also higher in patients with kidney damage without hypertension, proteinuria, or loss of kidney function, and are further modulated by these three elements. As expected, risks increase in the presence of pregestational hypertension and proteinuria and in proportion to reduction in kidney function [11,12,13].

Once a critical reduction in kidney tissue has been reached, a vicious circle is generated, leading to glomerular hypertension and hyperfiltration on the remnant nephrons, causing hypertrophy and ultimately proteinuria and glomerular sclerosis; proteinuria is further associated with glomerular sclerosis in a maladaptive vicious circle [14,15]. On account of this pathogenesis, the main approaches for the preservation of kidney function are aimed at reducing hyperfiltration and intraglomerular pressure and include angiotensin-converting enzyme inhibitors (ACEi) and angiotensin receptor blockers (ARBs), as well as diets with a reduced protein content [16,17].

The definition of low-protein diets has changed over time, in line with the changes in the definition of the recommended daily protein allowances, which are usually taken as a reference of a “normal” protein content in diets. Recommended daily allowances have progressively decreased to the current indication of 0.8 g of proteins per kg of body weight per day, and the current recent guidelines of the Kidney Disease Outcome Quality Initiative (K-DOQI) regarding nutritional management in CKD patients define low-protein diets as those with a protein content of 0.6 to 0.4 g/kg/day, and establish that the energy intake should be 25–35 kcal/kg of ideal body weight per day, according to age and metabolic needs [18].

In recent years there has been growing interest in plant-based diets, given their reduced phosphate content, lower induction of acidosis, and lower probability of triggering hyperfiltration compared to diets with the same amount of animal-derived proteins [19,20,21].

The recent K-DOQI guidelines on nutritional management in CKD patients underline the interest in plant-based diets, not only citing their potential advantages, but also clearly stating that the “there is insufficient evidence to recommend a particular protein type (plant vs. animal) in terms of the effects on nutritional status, calcium, or phosphorus levels, or the blood lipid profile.” This statement (3.2 Statement on Protein Type [18]) acknowledges that well-balanced plant-based diets are no longer considered as dangerous or inferior in CKD patients [18,19,20,21].

Plant-based diets are likewise an acknowledged option in pregnancy, provided that they are correctly followed, thus controlling for the complementarity of aminoacids and avoiding nutritional deficits, in particular those of vitamins (B12, vitamin D) and iron [22,23,24,25,26]. Furthermore, some data suggest that plant-based diets in pregnancy may even protect from the development of preeclampsia and hypertensive disorders of pregnancy, possibly through the prevention of excessive weight gain in pregnancy [25,26].

Pregnancy is a well-known cause of physiological renal hyperfiltration; an increase in glomerular filtration is also observed in CKD patients, where it is seen as a sort of “stress test” and is associated with an increase in proteinuria. Given the safety risks posed to the fetus, angiotensin converting enzyme inhibitors (ACEi) and angiotensin receptor blockers (ARBs) are discontinued, which further worsens proteinuria in CKD pregnancies [27,28].

In this context, our team and a few others tried to counterbalance pregnancy-induced hyperfiltration and reduce proteinuria by employing plant-based diets with moderately reduced protein intake during pregnancy [29,30,31,32,33].

The initial results were encouraging both in terms of fetal growth and period of delivery [29,30].

The aim of this study was to compare the results obtained in 52 CKD pregnancies, resulting in singleton deliveries, who followed a plant-based moderately protein-restricted diet during pregnancy, with a propensity-score-matched cohort of CKD pregnancies on unrestricted diets.

## 2. Materials and Methods

### 2.1. Settings of the Study

The present study was undertaken in two Italian centers, which began a conjoint study (TOCOS: Torino Cagliari Observational Study) on CKD and pregnancy in 2000: Turin (Piemonte, northern Italy), where the on-diet cases were followed, and Cagliari (the island of Sardinia) where the controls were selected [5].

In Turin, an industrial city with about 800,000 inhabitants, the study was performed at Ospedale Sant’Anna, a tertiary-care hospital that is part of the Città della Salute e della Scienza. With an average of 7000 deliveries per year, Sant’Anna is one of the largest European tertiary-care obstetric facilities. Since 2000, the center has run an outpatient facility for CKD pregnancies and acute kidney problems in pregnancy. The dietary approach with a moderately protein-restricted, plant-based diet has been employed since then [29,30].

The Azienda Ospedaliera Brotzu (AOB), Cagliari, is the largest hospital in Sardinia, an island with about 1.6 million inhabitants. The obstetric ward follows 800–1000 deliveries per year, offering care for high-risk pregnancies (thalassemia, diabetes, and kidney and autoimmune diseases). A nephrology outpatient service specializing in kidney diseases in pregnancy has been operative since 1995.

While both centers follow common protocols of control visits and care [28], the Cagliari center does not employ moderately protein-restricted, plant-based diets during pregnancy. For the sake of this study, the cases followed up in Torino were matched via a propensity score with controls selected in Cagliari, as subsequently specified.

### 2.2. Definitions Employed

Chronic kidney disease was defined according to the 2002 Kidney Disease Outcomes Quality Initiative (KDOQI) classification and stratification: estimated glomerular filtration rate (eGFR) < 60 mL/min/1.73 m^2^ for ≥ 3 months, or kidney damage for ≥ 3 months (Supplementary Table S1). The latter was defined as structural or functional anomalies of the kidneys, with or without decreased GFR, manifested either as pathological abnormalities or markers of kidney damage, including abnormalities in the composition of blood or urine, or abnormalities in imaging tests [1].

Since pre-pregnancy data were available for only a minority of the patients in the study, the definitions of stages were performed using data at referral.

Preeclampsia (PE) and hemolysis, elevated liver enzymes, and low platelets syndrome (HELLP) were defined according to the ACOG guidelines [34]. PE was diagnosed with hypertension (sBP ≥ 140 mm Hg and/or dBP≥90 mm Hg, on two occasions at least 4 h apart, with no underlying cause, in a woman with previously normal blood pressure), associated with proteinuria (24 h excretion ≥300 mg), diagnosed after 20 weeks of gestation or in the absence of proteinuria, with new onset of any of the following: platelet count <100,000/μL, serum creatinine >1.1 mg/dl, or doubling of its concentration in absence of other renal disease, transaminases to twice normal concentration of liver enzymes, pulmonary edema, cerebral/visual symptoms [34,35].

HELLP syndrome was defined in accordance with the above guidelines (alanine or aspartate transaminase levels ≥ twice the upper limit of normal; lactate dehydrogenase ≥ 600 U/L; platelet count <100,000/μL) [34]. Superimposed preeclampsia was defined as preeclampsia on already-known treated or untreated pregestational hypertension, or on already-known CKD [35].

Small-for-gestational-age babies were defined in accordance with the two most commonly used cut-points: below the 5th and 10th centiles, following INTERGROWTH standards [36].

Preterm delivery was defined as delivery before 37 completed gestational weeks; early preterm delivery as before 34; and very early preterm delivery as delivery before 28 completed gestational weeks.

Obesity was defined as a pregestational body mass index (BMI) equal to or above 30 kg/m^2^; overweight was defined as BMI between 25 and 30 kg/m^2^; and underweight was defined as pregestational BMI < 20 kg/m^2^.

Gestational hypertension was defined in line with the current guidelines [34,35,36,37,38].

Antihypertensive treatment employed a combination of alphamethyl-dopa and nifedipine, adding doxazosin and small doses of diuretics or clonidine when needed. Treatment was adjusted at every clinical visit with a target of 120–130/60–70 mm Hg [28]. In both settings of care, the frequency of nephrology and obstetric visits, blood and urine tests and biometric and Doppler studies of the uterine and umbilical arteries were tailored to individual needs (visits: 1 week–1 month apart, biometry and fetal Doppler according to fetal growth and the presence of Doppler anomalies), in keeping with the Italian best practices in pregnant CKD patients [28].

Low-dose acetylsalicylate was increasingly prescribed, first to patients with severe proteinuria, and more recently to all women with at-risk pregnancies.

### 2.3. Indications for the Diet

The main indications for plant-based diets in pregnancy were progressively broadened from subjects with CKD Stages 4–5 and/or nephrotic syndrome to include: patients already on a plant-based diet before pregnancy; CKD Stage 3 to 5 patients not on dialysis; CKD Stage 1 and 2 patients with kidney function impairment during pregnancy; proteinuria above 3 g/day at any time (<30 gestational weeks) of pregnancy, or proteinuria above 1 g/day at referral or in the first trimester; previous nephrotic syndrome, increase in or development of proteinuria in pregnancy; or a combination of any of these elements. The following contraindications were considered: anorexia, hyperemesis gravidarum, language barriers impairing understanding the diet and its aim, psychiatric disorders, low compliance to prescriptions and controls.

### 2.4. Selection of the Control Group

In line with the criteria used to prescribe a plant-based diet, we selected the controls in the TOCOS database solely for patients being followed up in Cagliari.

The patients of the control group did not undergo a specific nutritional workout in pregnancy, and their protein intake was unrestricted. Patients were referred to the dieticians in the case of excessive weight gain or in the case of hyperemesis. Their follow-up is in keeping with the usual indications of good clinical practice [28,39].

The control patients were chosen using a propensity-match score, based on CKD stage (1, 2, 3, 4, and 5 considered together) and level of proteinuria, dichotomized at 1 g at the first clinical control visit during pregnancy.

### 2.5. The Plant-Based, Moderately Protein-Restricted Diet

The plant-based diet is based on a simplified schema.

Food is chosen according to a qualitative approach (allowed/forbidden) and the patients are not obliged to regularly weigh food. The dietician controls the quality, quantity, and integration of proteins every four to eight weeks, based upon a 5-day food diary. The use of food diary (5 days instead of the 3 recommended ones, according to the KDOQI guidelines, to improve the quality of the reporting) is chosen, in the absence of other agreed methods, to indirectly evaluate nutrient intake in pregnancy. The frequency of the dietary controls is personalized, according to the clinical status, and the dietitian sees the patients in the occasion of their conjoint nephrology and obstetric control.

When the diet is first prescribed, a short-term consultation (1–2 weeks) is organized, either in person or by phone. Subsequently, the frequency ranges from every 8 weeks in stable patients, with regular weight gain and fetal growth, who follow the diet without declaring difficulties, doubts or problems; to every 2–4 weeks, in particular in cases with either no weight gain, or important weight gain in which—as it is usual in pregnancy—the discrimination between lean weight and fluid overload is not simple, in the absence of overt oedema, and without being able to rely on bioimpedance, whose use is not standardized in pregnancy.

The goal is to control protein intake to 0.8 g/kg/day (real weight) with over 80% of protein coming from plant-derived sources. In normal individuals, 0.8 g/kg/day of proteins is now considered the reference intake (daily allowances); in pregnancy, in patients on plant-based diets, it is advised to increase by 20%, i.e., to 1 g/kg/day [25]. In CKD patients, the recommended intake of 0.6 g/kg/day, taking as reference the recent K-DOQI guidelines, was likewise increased; to be on the safe side, the increase was established at around 30% (rounded at 0.8 g/kg/day) [28,29,30].

While the overall approach remained the same throughout the period of study, partly on account of changes in definitions of diets with little or no animal-derived proteins in the overall population, we now use the term “plant-based” rather than “vegan-vegetarian” [28,29,30,40].

The most recent version of the diet is reported in the supplementary material.

The energy intake followed the usual indications in pregnancy. In accordance with the current recommendations, the target was a weight increase ranging between 11.5 kg and 16 kg, adapted for underweight and overweight women [41]. The energy intake followed the usual indications in pregnancy. No variation was advised in plant-based diets in pregnancy [25]. Based on the kidney function, proteinuria levels, and the individual patient’s needs and preferences, we allowed 1–3 unrestricted meals per week (without protein restriction but limited in unsaturated fats and short-chain sugars, as indicated in pregnancy). To facilitate compliance with a plant-based diet without the need to eat pulses and cereals at each meal, as it is necessary to avoid deficits of specific aminoacids (in particular lysine), in most cases, supplementation of alpha-keto analogues and aminoacids (Alpha-Kappa or Ketosteril) was added (1 tablet per each 8–10 kg of pregestation body weight) [28,29,30,31,32,33]. This choice was indeed made to remain “on the safe side” and to avoid the risk of deficits of specific aminoacids in patients who were not used to a plant-based regiment before pregnancy. However, in some patients in which the pill burden was felt to be too high or the supplementation was not tolerated (gastrointestinal discomfort, 1 case) a standard plant-based diet, or a lower pill number, was prescribed, under strict nutritional surveillance [42].

The food distribution and the choice of specific food is highly individualized. For example, for energy intake, a wide choice is discussed, including choosing among the main sources of long-chain carbohydrates; bread and pasta, the bases of the Italian cuisine, are usually widely employed. However, rice, couscous, potatoes, sweet potatoes, and polenta may be either occasional alternatives or the basis of the diet for patients who prefer them.

Olive oil, once more in line with the Mediterranean cuisine and widely available in our country, is preferred; butter and margarine are avoided, but sunflower oil or colza oil are occasionally used by patients who do not like olive oil. Likewise, the choice between the pulses is personalized, and all efforts are made to make the “plant-based” menu as varied as possible. Furthermore, the allowance of unrestricted meals, likewise with personalized frequency, increases the variety, and once again, the choice between meat, fish, poultry, eggs, or cheese, or a combination of animal-derived food, is left free in order to also reduce the psychological constrain of such a demanding diet.

An example of a plant-based diet used in our setting is shown in Supplementary Table S4.

Given the lack of indications on salt restriction in CKD pregnancy, we did not restrict salt intake, but we recommended moderate sodium reduction to patients with severe edema.

Iron status, B12, and 25-OH vitamin D were controlled at baseline and up to monthly tests if needed; vitamins and iron supplements were employed on the basis of blood test results. Erythropoietin was used when needed, with a hemoglobin target of 10 g/dL on account of the physiological hemodilution found in pregnancy.

### 2.6. Statistical Evaluation

Source of data: in each setting, data on all patients referred with known, newly-diagnosed, or suspected CKD in pregnancy were prospectively recorded in dedicated databases, which were periodically merged, with a final coherence control performed by a trained statistician. Prescription of the moderately protein-restricted plant-based diet is recorded in the database. Data about multiple pregnancies and miscarriages and pregnancy terminations were gathered, but not considered in the statistical analysis. The full list of data gathered is available on request.

The propensity match considered proteinuria (dichotomized at 1 g at baseline) and CKD stage, by means of a greedy 1:1 algorithm. Matching was performed using the “Matchit” R package v 4.2.0 [43].

Continuous series were tested for normality using the Shapiro–Wilk test, and homoscedasticity with Leven’s test. According to the conditions of application, for comparing two groups (e.g., seen in nephrology, not seen in nephrology), the independent Student *t*-test and Wilcoxon rank-sum test were used. To compare 3 or more groups, one-way ANOVA and the Kruskal–Wallis test were used.

The comparison of proportions was made with the Chi-squared or the Fisher test, depending on the size of subsample involved. Results are displayed with the median and the interquartile range (IQR, or Q1–Q3 quartiles) or as mean and standard deviation, as appropriate.

The following outcomes were tested by univariable and multivariable methods: birth centile <10 and <5, preterm delivery (all: <37, early: <34 and very early: <28 complete gestational weeks); the outcomes were combined into severe (birth weight<5 centile or delivery <28 weeks) and general (birth weight<10 centile or preterm delivery <37 weeks). The choice of the covariates to include in the multivariate model was based on either statistical significance at the univariate analysis, or well-acknowledged clinical relevance, for instance, diet (plant-based vs. unrestricted); CKD stage (1, 2, 3, 4, and 5 considered together); proteinuria at the first control visit during pregnancy (<1 g vs. ≥1 g/24 h), and hypertension (present/absent).

The model employed a backward deletion method and standardized residuals were verified.

Temporal series (e.g., weeks at delivery) were visually analyzed using inverse Kaplan–Meier curves and differences were tested using the log-rank test.

The statistical analysis was performed with JASP version 0.14.1 (Armstrong, The Netherlands, EU) and RStudio version 3.3.0 (Rstudio Project, Boston, MA, USA).

Alpha error was fixed at 5%.

### 2.7. Ethical Issues

This observational study on current clinical practice was approved by the Ethics Committee of the OIRM Sant’Anna (n° practice 335; n° protocol 11551/c28.2 del 4/3/2011). All patients signed a dedicated informed consent at the first control visit during pregnancy.

Availability of data and materials: TOCOS is a dynamic database updated in real time; the most recent update can be obtained by sending a motivated request to the contact author.

## 3. Results

### 3.1. Baseline Data

**Figure 1:**
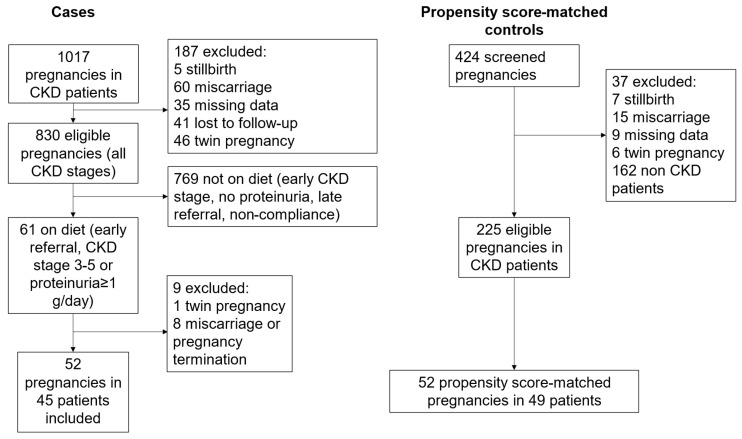
Study flow chart.

The 52 pregnancies on the plant-based diet considered for the analysis were selected from 61 pregnancies, after the exclusion of one twin pregnancy, and of 8 cases with miscarriages or pregnancy terminations (the flow chart of the study is reported in Figure 1).

Table 1 reports the baseline data in the propensity-matched cohorts selected from the Cagliari database.

Even though the matching considered only CKD stage and proteinuria, the two cohorts were perfectly matched for all the parameters considered, except for ethnicity (non-Caucasian ethnicity was more frequent in Torino, a large industrial city, than in Cagliari, a smaller city in a prevalently agricultural area). In both settings, more than half of the patients were affected by glomerulonephritis, and over one-third were in CKD Stage 3 or higher at their first control visit during pregnancy.

### 3.2. Pregnancy Outcomes, According to Diet Prescription

Table 2 reports on the main pregnancy outcomes found in the two groups (on diet/not on diet).

While a tendency towards longer pregnancy duration was observed in on-diet patients (preterm delivery: 42.31% on diet vs. 28.85% control group), statistical significance was reached only for delivery <28 weeks (5.77% on-diet patient vs. 11.54% controls, *p* = 0.012).

The combined general outcome (birth before 37 completed gestational weeks and birth centile <10) was observed in 61.54% of the pregnancies in the on-diet group versus 80.77% in the control group (*p* = 0.030).

No on-diet patient and no patient in the control group died, and no mother in either group started dialysis in the first 3 months after delivery. Three neonatal deaths were recorded in the control group, all linked to prematurity, while none were observed in the on-diet group.

None of the surviving children had severe malformations: no malformations were recorded in the control group, while in the on-diet group two newborns presented interatrial septal defects, linked to prematurity, which in both cases spontaneously resolved; and one baby presented ankyloglossia. In addition, one case of complex cardiac anomaly was recorded in a twin pregnancy (excluded from the present analysis) in the on-diet group; the mother was affected by type 1 diabetes, and the child died in the first week of life.

**Figure 2:**
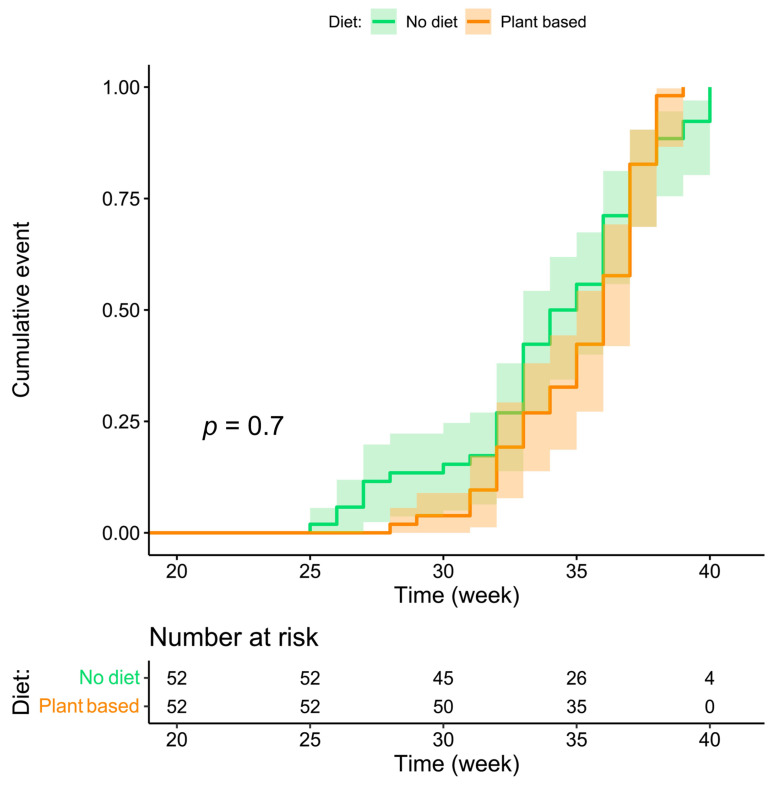
Kaplan–Meier curve for week of delivery according to the patient’s diet.

The Kaplan–Meier curve of timing of delivery according to whether or not a diet was prescribed is shown in Figure 2. The curve supports the noninferiority of plant-based diets for the duration of gestation and seems to indicate that they contribute to reducing the incidence of early preterm delivery.

### 3.3. Variations in Proteinuria and eGFR from the First to Last Control Visit during Pregnancy

**Figure 3:**
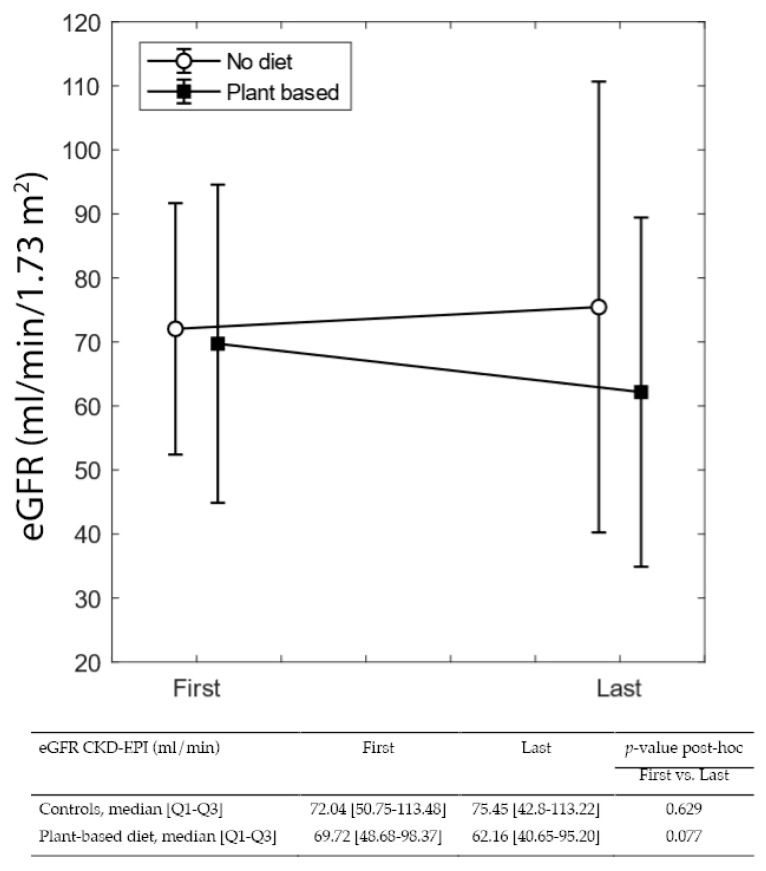
eGFR at the first and last control visit in pregnancy.

**Figure 4:**
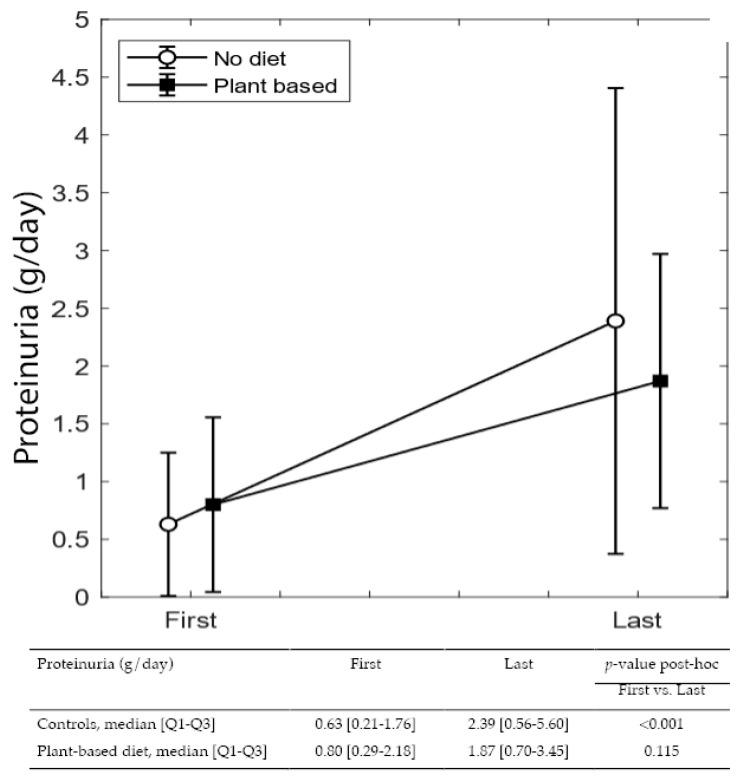
Proteinuria variation during pregnancy.

Figure 3 and Figure 4 summarize differences in eGFR and proteinuria from referral to delivery, in on-diet cases, and controls. The single-patient graphs are available in Supplementary Figure S1.

While differences from referral to the last control visit during pregnancy were not significant in either group, the increase in proteinuria from the first to the last visit is statistically significant in controls (from 0.63 to 2.39 g/24 h *p* < 0.0001), but nonsignificant in patients on plant-based diets, despite a higher baseline value (median from 0.80 to 1.87 g/24 h).

### 3.4. Logistic Regression Analysis

The univariable analysis for the different outcomes (preterm delivery and small for gestational age newborns) is reported in the supplementary material (Table S2).

Following the plant-based diet was associated with a lower incidence of all adverse outcomes, which was statistically significant for early preterm delivery (delivery before 34 completed gestational weeks (Table 3); statistical significance was also reached for two general combined outcomes (delivery before 37 gestational weeks or centile <10 and delivery before 34 gestational weeks or centile <10), with a strong protective effect of having been on a plant-based diet (delivery before 37 gestational weeks or centile <10: OR: 0.260 [0.093–0.724], *p* = 0.010) found after adjustment for stage, proteinuria, and hypertension (Table S4). Following a plant-based diet was instead not associated with the single outcome centile <10 or centile <5, delivery before 37 or 28 completed gestational weeks or with the combined outcomes of delivery before 34 gestational weeks or centile <5 and delivery before 28 gestational weeks or centile <5 (Supplementary Table S3).

## 4. Discussion

The main result of this study is to highlight the potential benefits of plant-based, moderately protein-restricted diets in pregnancy for patients with forms of CKD at high risk of adverse pregnancy outcomes, characterized either by a reduction in kidney function, a high level of proteinuria at baseline, or both.

While previous studies already suggested that such a nutritional approach is safe and is associated with better pregnancy outcomes, the limits of the previous analyses were linked to the small number of cases studied and the lack of a well-matched control group [29,30,31,32,33]. This is the first time that we are reporting the results of a study involving a larger group of on-diet patients, compared with a control group that was propensity-matched for the two main elements considered for diet prescription (CKD stage and proteinuria). It should be noted that the two matched groups of 52 patients each were superimposable for age, BMI, prevalence of primiparity, type of disease, and week of referral. The only difference (higher prevalence of women of non-Caucasian origin in the on-diet group) reflects demographic differences between the two settings (Table 1).

The results suggest that on-diet patients have an advantage for the main outcomes considered (preterm delivery and small-for-gestational-age babies), which reaches statistical significance for the general combined outcome (delivery at <37 completed gestational weeks and birth centile <10), which occurred in 61.54% of on-diet patients and in 80.77% of controls (*p* = 0.030) (Table 2). The multivariable analysis, after adjustment for the main potential confounders (CKD stage, hypertension, and proteinuria), indicates an odds ratio of 0.260 (0.093–0.724) of reaching the combined outcome (weeks < 37 or centile < 10) for on-diet patients (Table 3). The visual analysis of the Kaplan–Meier curve suggests that the advantage occurs early in gestation: no case of delivery <28 gestational age was recorded in the on-diet group, while the prevalence of delivery <34 weeks was 26.92% in the on-diet group and 42.31% in controls (Table 2, Figure 2).

In both cases and controls, probably as a reflection of diligent follow-up targeting normalization of blood pressure and avoiding excessive weight gain, eGFR remained stable from referral to the last check-up before delivery. Instead, proteinuria increased sharply in controls (from 0.63 to 2.39 g/24 h; *p* < 0.001), while the increase was far lower in on-diet patients (0.8 to 1.87 g/24 h; *p* = 0.115) (Figure 2 and Figure 3).

While the small, but potentially relevant, gain in duration of gestation (36 weeks in on-diet patients versus 34.5 in controls) and in birth centile (38.81 in on-diet patients versus 32.84 in controls; Table 2) is not statistically significant, possibly due to the small number of cases, the difference in proteinuria is significant, and suggests less hyperfiltration on the remnant nephrons, which is in turn associated with lower oxidative stress and endothelial damage [14,19,21,44]. Furthermore, plant-based diets are rich in antioxidants and are less diabetogenic in pregnancy [23,24,25]. All these “endothelial protecting effects” may also play a role in preserving placental function, often impaired in CKD, as has been highlighted in the most recent guidelines on nutrition in CKD [18].

It is difficult to discuss our data in the context of other studies on diets in CKD pregnancies, since, as recently reviewed, there is a cruel lack of such information, specifically in the predialysis phase [45,46]. Plant-based diets are, however, increasingly advised in other situations at risk for adverse pregnancy outcomes that also share a higher risk for endothelial damage, including diabetes, hypertension, and obesity. Of note, all these conditions are risk factors for the development of chronic kidney disease, and may be associated with subclinical renal damage, hence suggesting that common factors are at play in this situations, possibly including—besides endothelial protection—modulation of gut microbiota, and avoidance of excessive weight gain [25,47,48].

More specifically regarding the kidney function, at the same levels of protein intake, plant-based diets are known to induce a lower degree of hyperfiltration compared to mixed protein diets [19,20,21]. Accordingly, moderate protein restriction, as in our diet plan, in which the diet recommended in pregnancy corresponds to the indications for a healthy, nonpregnant individual, the plant-based regimen is expected to blunt pregnancy-induced renal hyperfiltration.

The potential implications of our results are wide: if the advantage in CKD pregnancies is confirmed on a larger scale, then plant-based diets, with a protein intake adapted to the degree of kidney function and proteinuria, could become the standard indication for women with CKD in pregnancy, at least in severe cases, which is estimated as present in 1:500–1:1000 pregnancies. Furthermore, our experience further underlines the interest of plant-based diets in other high-risk pregnancies, including pre-existing diabetes and hypertension.

This study, which has the advantage of reporting on one treatment proven to be beneficial in CKD pregnancies, and of correlating it for the first time with a lower increase in proteinuria during gestation, has several limitations. First, it involves only two centers. Secondly, as the data were gathered retrospectively, over a long period of time, we were not able to control for variations in care, including pharmacologic treatments, occurring during that period. However, as the Italian best practices in pregnancy in CKD were based on care at these two centers, this is evidence of concordant approaches [28]. Furthermore, although this is the largest CKD cohort followed up with plant-based diets in pregnancy so far published, the number of patients involved is still relatively small, and further stratification, for example, concerning kidney disease, was not possible. The lack of specific nutritional management in CKD pregnancies in the control group did not allow for a detailed comparison of the diet composition to be performed; however, the patients in Cagliari were followed up according to the usual standard of care, in the context of a predominantly Mediterranean nutrition pattern [49,50].

Lastly, and once more due to the only recent introduction in clinical practice of placental biomarkers, the levels of s-flt1 and PlGF were not available, neither for cases nor controls.

Future research is absolutely needed in the field of nutrition in CKD pregnancies. While randomized controlled studies are difficult to propose in the delicate and heterogeneous population with CKD in pregnancy, we need at least large cohort studies comparing different dietary patterns and approaches in these patients. Analysis of placental morphology and correlation with placental biomarkers in CKD patients, as well as in comparison with other diseases sharing similar challenges, such as hypertension, diabetes, and morbid obesity, may allow for a better understanding of the protective effects exerted by plant-based diets, and help us to more finely tune prescriptions.

## 5. Conclusions

Plant-based, moderately protein-restricted diets are feasible in pregnancy in patients with CKD, and are associated with a lower risk of preterm delivery and of delivery of small-for-gestational-age babies, as compared to mothers on unrestricted omnivorous diets. The favorable effect observed might be mediated by a better stabilization of proteinuria throughout pregnancy, probably resulting from a lower hyperfiltration challenge.

Further prospective studies, systematically employing biomarkers of placental health as well as exploring placental pathology in CKD patients on different diets, are needed if we are to better understand the reasons for these promising results, so that plant-based diets can eventually be proposed also in other high-risk pregnancies.

## Figures and Tables

**Table 1:** Baseline data in CKD pregnancies, according to prescription of a plant-based diet.

	All	No Diet	Plant-Based Diet	*p*-Values
**Overall data CKD**				
*N*	104	52	52	
**Baseline data**				
Age (years), median [Q1–Q3]	34 [31.75–38]	34.5 [33–38]	34 [30.75–38]	0.533
Parity (primiparous), *n* (%)	66 (63.46%)	36 (69.23%)	30 (57.69%)	0.222
BMI (kg/m^2^), median [Q1–Q3]	23.15 [20.9–26.62]	22.9 [20.19–26.04]	23.63 [21.48–26.62]	0.485
BMI ≥ 30 kg/m^2^, *n* (%)	14 (13.46%)	7 (13.46%)	7 (13.46%)	1
Ethnicity (non-Caucasian), *n* (%)	95 (91.35%)	1 (1.92%)	8 (15.39%)	**0.015**
**Baseline kidney function data**				
Serum creatinine, median [Q1–Q3]	1.02 [0.73–1.39]	0.99 [0.65–1.31]	1.04 [0.79–1.41]	0.301
eGFR CKD-EPI (mL/min), median [Q1–Q3]	71.21 [40.42–106.40]	72.04 [50.75–113.48]	69.72 [48.68–98.37]	0.435
Stage 1, *n* (%)	31 (29.81%)	16 (30.77%)	15 (28.85%)	0.514
Stage 2, *n* (%)	33 (31.73%)	16 (30.77%)	17 (32.69%)	
Stage 3, *n* (%)	19 (18.27%)	11 (21.15%)	8 (15.39%)	
Stage 4, *n* (%)	15 (14.42%)	8 (15.39%)	7 (13.46%)	
Stage 5, *n* (%)	6 (5.775)	1 (1.92%)	5 (9.62%)	
Proteinuria (g/24 h), median [Q1–Q3]	0.705 [0.24–2.06]	0.63 [0.21–1.76]	0.80 [0.29–2.18]	0.196
Proteinuria < 0.5 g/24 h, *n* (%)	38 (36.54%)	20 (38.46%)	18 (34.62%)	0.739
Proteinuria 0.5–1 g/24 h, *n* (%)	24 (23.08%)	12 (23.08%)	12 (23.08%)	
Proteinuria 1–3 g/24 h, *n* (%)	26 (25%)	14 (26.92%)	12 (23.08%)	
Proteinuria ≥ 3 g/24 h, *n* (%)	16 (15.39%)	6 (11.54%)	10 (19.23%)	
**Timing of referral**				
Week at referral, median [Q1–Q3]	8 [6–12]	9 [7–12.25]	7.5 [6–12]	0.201
<12 gestational weeks, *n* (%)	73 (70.195)	36 (69.23%)	37 (71.15%)	0.757
13–23 gestational weeks, *n* (%)	8 (7.69%)	5 (9.62%)	3 (5.77%)	
≥24 gestational weeks, *n* (%)	23 (22.12%)	11 (21.15%)	12 (23.08%)	
**Cause of CKD**				
Glomerular (primary and secondary GN), *n* (%)	54 (51.92%)	27 (51.92%)	27 (51.92%)	1
Single kidney, *n* (%)	3 (2.89%)	2 (3.85%)	1 (1.93%)	0.558
Diabetic nephropathy, *n* (%)	14 (13.46%)	5 (9.62%)	9 (17.31%)	0.250
ADPKD, *n* (%)	6 (5.77%)	4 (7.69%)	2 (3.85%)	0.400
Kidney graft, *n* (%)	9 (8.65%)	5 (9.62%)	4 (7.69%)	0.727
Interstitial (includes interstitial nephropathies, kidney stones, CAKUT and urologic malformations), *n* (%)	10 (9.62%)	5 (9.62%)	5 (9.62%)	1
Other, *n* (%)	8 (7.96%)	4 (7.69%)	4 (7.69%)	1

Legend: N, cohort size; BMI, body mass index; eGFR, estimated glomerular filtration rate; GN, glomerulonephritis; ADPKD, autosomal dominant polycystic kidney disease; CAKUT, congenital anomalies of the kidneys and urinary tract; APN, acute pyelonephritis. In bold, significant differences.

**Table 2:** Main outcomes according to the diet prescribed.

	All	No Diet	Plant-Based Diet	*p*-Values
**Overall data CKD**				
*N*	104	52	52	
**Renal data at last control visit**				
Serum creatinine, median [Q1–Q3]	1.1 [0.74–1.64]	1 [0.7–1.56]	1.18 [0.81–1.65]	0.194
Proteinuria (g/24 h), median [Q1–Q3]	1.97 [0.58–4.46]	2.39 [0.56–5.6]	1.87 [0.70–3.45]	0.338
eGFR CKD-EPI (mL/min), median [Q1–Q3]	65.62 [41.66–105.01]	72.93 [42.8–113.22]	62.16 [40.65–95.20]	0.302
Stage shift (increase of at least 1 CKD stage), *n* (%)	21 (20.19%)	11 (21.15%)	10 (19.23%)	0.807
**Delivery**				
Week of delivery, median [Q1–Q3]	36 [33–37]	34.5 [32–37]	36 [33–37]	0.164
Term ≥ 37 gw, *n* (%)	37 (35.58%)	15 (28.85%)	22 (42.31%)	0.152
Term < 34 gw, *n* (%)	36 (34.62%)	22 (42.31%)	14 (26.92%)	0.099
Term < 32 gw, *n* (%)	14 (13.46%)	9 (17.31%)	5 (9.62%)	0.250
Term < 28 gw, *n* (%)	6 (5.77%)	6 (11.54%)	0	**0.012**
**Offspring data**				
Weight at delivery, median [Q1–Q3]	2380 [1797–2820]	2350 [1737.5–2727.5]	2537.5 [1957.5–2872.5]	0.254
Weight < 2500 g, *n* (%)	54 (51.92%)	29 (55.77%)	25 (48.08%)	0.432
Weight < 1500 g, *n* (%)	15 (14.42%)	10 (19.23%)	5 (9.62%)	0.163
Centile, median [Q1–Q3]	36.30 [9.45–59.03]	32.84 [6.29–57.33]	38.81 [14.74–62.08]	0.270
Centile < 10, *n* (%)	28 (26.92%)	18 (34.62%)	10 (19.23)	0.077
Centile < 5, *n* (%)	19 (18.27%)	12 (23.08%)	7 (13.46%)	0.205
**Pregnancy-related outcomes**				
PE, *n* (%)	3 (2.89%)	3 (5.77)	0	0.079
**Combined outcomes**				
Term < 37 gw or Centile < 10, *n* (%)	74 (71.15%)	42 (80.77%)	32 (61.54%)	**0.030**
Term < 34 gw or Centile < 10, *n* (%)	50 (48.08%)	29 (55.77%)	21 (40.39%)	0.116
Term < 34 gw or Centile < 5, *n* (%)	43 (41.35%)	24 (46.15%)	19 (36.54%)	0.319
Term < 28 gw or Centile < 5, *n* (%)	20 (19.23%)	13 (25%)	7 (13.46%)	0.135

Legend: N, cohort size; eGFR, estimated glomerular filtration rate; PE, preeclampsia; gw, gestational week. In bold, significant differences.

**Table 3:** Multivariable logistic regression.

Preterm Delivery: Gestational Weeks <34			
		**OR [CI 95%]**	*p* Value
**First step**	CKD stage	1.495 [0.928–2.406]	0.098
	**Plant-based diet**	**0.320 [0.122–0.843]**	**0.021**
	Proteinuria > 1 g	1.761 [0.698–4.443]	0.231
	**Hypertension**	**5.739 [2.180–15.106]**	**<0.001**
**Last step**	CKD stage	1.455 [0.909–2.328]	0.118
	**Plant-based diet**	**0.336 [0.129–0.873]**	**0.025**
	**Hypertension**	**5.697 [2.188–14.837]**	**<0.001**
**Combined Outcome: Preterm Delivery at Week <37 or <10 Centile**			
		**OR [CI 95%]**	*p* value
**One step only**	**CKD stage**	**2.685 [1.494–4.828]**	**<0.001**
	**Plant-based diet**	**0.260 [0.093–0.724]**	**0.010**
	Proteinuria > 1 g	2.720 [0.936–7.905]	0.066
	Hypertension	2.294 [0.827–6.367]	0.111
**Combined Outcome: Preterm Delivery at Week <34 or <10 Centile**			
**One step only**	CKD stage	1.460 [0.935–2.280]	0.096
	**Plant-based diet**	**0.383 [0.158–0.928]**	**0.034**
	Proteinuria > 1 g	2.118 [0.879–5.104]	0.094
	**Hypertension**	**4.056 [1.679–9.798]**	**0.002**

In bold, significant values.

## Data Availability

The data presented in this study are available on request from the corresponding author.

## Supplementary Materials

The following are available online at https://www.mdpi.com/article/10.3390/nu14194203/s1, Table S1: definition and stages of chronic kidney disease according to Kidney Disease Outcome Quality Initiative (KDOQI) guideline 2002; Table S2: univariate logistic regression for different outcomes; Table S3: multivariable logistic regressions for different outcomes; Table S4: The rationale of the plant-based diet the diet and an example for one day of plant-based meals and further advice for pregnant women; Figure S1: eGFR and proteinuria at referral and delivery. First point, referral; second point, delivery.
