# Supplementary material for: Plant-Based Diets Improve Maternal–Fetal Outcomes in CKD Pregnancies
Source: Nutrients. 2022 Oct 9;14(19):4203. doi: 10.3390/nu14194203 (PMC9573150; doi:10.3390/nu14194203)

Supplementary material:

Table S1: *definition and stages of Chronic Kidney Disease according to Kidney Disease Outcome Quality Initiative (KDOQI) guideline 2002*

**STAGE**	**DESCRIPTION**	**GFR (mL/min/1.73 m2)**
1	Kidney damage with normal GFR	≥ 90
2	Kidney damage with mild reduction in GFR	60-89
3	Moderate reduction in GFR	30-59
4	Severe reduction in GFR	15-29
5	Kidney failure	< 15 or dialysis

Legend: GFR, glomerular filtration rate

Table S2: *univariate logistic regression for different outcomes*

	Outcomes					
	Centile <10		Centile <5			
	OR [CI 95%]	P value	OR [CI 95%]]	P value		
HTA	1.425 [0.597-3.400]	0.424	1.013 [0.374-2.742]	0.981		
CKD stage	1.074 [0.695-1.659]	0.749	1.206 [0.732-1.986]	0.462		
Diet	0.450 [0.184-1.101]	0.080	0.519 [0.186-1.445]	0.209		
PTO >1g	2.564 [1.057-6.218]	**0.037**	0.418 [0.152-1.149]	0.091		
	Outcomes					
	Preterm <37w		Preterm <34w		Preterm <28	
	OR [CI 95%]]	P value	OR [CI 95%]	P value	OR [CI 95%]]	P value
HTA	2.144 [0.936-4.909]	0.071	5.087 [2.098-12.333]	**<0.001**	2.356 [0.412-13.464]	0.335
CKD stage	2.596 [1.582-4.260]	**<0.001**	1.527 [1.006-2.318]	**0.047**	0.865 [0.372-2.011]	0.736
Diet	0.553 [0.245-1.248]	0.154	0.502 [0.220-1.145]	0.101	-	-
PTO >1g	1.689 [0.729-3.913]	0.221	1.539 [0.678-3.492]	0.302	1.513 [0.290-7.882]	0.623
	Outcomes					
	<37w or <10 centile		<28w or <5 centile			
	OR [CI 95%]]	P value	OR [CI 95%]	P value		
HTA	2.745 [1.111-6.785]	**0.029**	1.154 [0.435-3.061]	0.774		
CKD stage	2.465 [1.463-4.153]	**<0.001**	1.297 [0.794-2.121]	0.299		
Diet	0.381 [0.157-0.925]	**0.033**	0.467 [0.169-1.286]	0.141		
PTO >1g	1.878 [0.759-4.645]	**0.173**	2.090 [0.779-5.602]	0.143		

Legend: HTA, hypertension; CKD, chronic kidney disease; PTO, proteinuria 24h

Table S3: *multivariable logistic regressions for different outcomes*

OUTCOMES

Centile <10

		OR [CI 95%]	P value
**First step**	CKD stage	1.099 [0.692-1.746]	0.688
	**Diet**	**0.387 [0.151-0.994]**	**0.048**
	**PTO>1g**	**2.787 [1.113-6.980]**	**0.029**
	HTA	1.511 [0.596-3.830]	0.384
**Last step**	**Diet**	0.414 [0.164-1.044]	0.062
	**PTO>1g**	**2.754 [1.109-6.838]**	**0.029**

Centile <5

		OR [CI 95%]	P value
**First step**	CKD Stage	1.270 [0.759-2.125]	0.363
	Diet	0.476 [0.165-1.375]	0.170
	PTO>1g	2.593 [0.922-7.289]	0.071
	HTA	0.978 [0.343-2.788]	0.967
**Last step**	PTO>1g	2.395 [0.870-6.590]	0.091

Birth at gestational weeks <37

		OR [CI 95%]	P value
**First step**	**CKD stage**	**2.796 [1.625-4.813]**	**<0.001**
	Diet	0.413 [0.163-1.050]	0.063
	PTO>1g	2.382 [0.895-6.343]	0.082
	HTA	1.655 [0.650-4.214]	0.291
**Last step**	CKD stage	2.954 [1.723-5.066]	**<0.001**
	Diet	0.429 [0.171-1.080]	0.072
	PTO>1g	2.458 [0.930-6.501]	0.070

Birth at gestational weeks <28

		OR [CI 95%]	P value
**First step**	CKD stage	0.796 [0.3317-1.913]	0.610
	PTO>1g	1.776 [0.307-10.269]	0.521
	HTA	3.259 [0.531-19.991]	0.202
**Last step**	HTA	2.356 [0.412-13.464]	0.335

Birth at gestational weeks <34 or Centile <5

		OR [CI 95%]	P value
**First step**	**CKD stage**	1.649 [1.057-2.575]	**0.028**
	Diet	0.504 [0.210-1.211]	0.126
	PTO>1g	1.728 [0.722-4.139]	0.219

Birth at gestational weeks <28 or Centile <5

		OR [CI 95%]	P value
**First step**	CKD stage	1.358 [0.815-2.262]	0.241
	Diet	0.418 [0.146-1.197]	0.104
	PTO>1g	2.295 [0.832-6.330]	0.109

	**HTA**	**3.424 [1.433-8.182]**	**0.006**		HTA	1.124 [0.402-3.141]	0.824
**Last step**	**CKD stage**	**1.600 [1.034-2.475]**	**0.035**	**Last step**	Diet	0.444 [0.159-1.243]	0.122
	Diet	0.520 [0.218-1.240]	0.140		PTO>1g	2.199 [0.807-5.992]	0.123
	**HTA**	**3.451 [1.454-8.191]**	**0.005**				

Legend: CKD, chronic kidney disease; PTO, proteinuria 24h; HTA, hypertension;

__Table S4:__ The rationale of the plant-based diet the diet and an example for one day of plant-based meals and further advice for pregnant women

Rationale of the diet

This diet is based on some simple assumptions:
- a low-protein diet is a "metabolic stabilizer".
- proteins of vegetable origin induce a lesser "workload" for the kidneys, and therefore are better stabilize renal function and reduce pregnancy-related hyperfiltration, which in turn is one of the elements that can increase loss of protein (proteinuria).
- the diet should allow a good quality of life and should be followed with flexibility, gradually adapting to the preferences of each person.
- during pregnancy the kidneys work harder than usual and some of the drugs that can normally be taken to allow the kidney to rest, cannot be used. For this reason, a period of dieting makes it possible to "gain time", in a pregnancy that is at risk either because of a reduction in kidney function or because there is significant proteinuria (which in turn increases the workload for sick kidneys).
- In pregnancy, we need to ensure that adequate levels of several nutrients, including vitamins and minerals (vitamin B12, folic acid, vitamin D, calcium), are maintained.

The experience of the people who follow a vegan-vegetarian diet by choice, not because of the presence of kidney disease, has demonstrated that such a diet, under nutritional control, is safe in pregnancy.
There are very few studies in the medical literature on low-protein diets in pregnant CKD patients and our experience, although obtained in a limited number of cases, it is one of the few available. To date the results have been positive, thanks in part to close collaboration between nephrologists and dieticians and to good patient compliance.

Our diet is based on eating a wide variety of foods of vegetable origin (cereals, pasta, bread, legumes, vegetables, fruit), usually integrated with supplements of amino acids and keto-acids, to further increase its nutritional safety, in particular in patients who have not previously followed a vegan-vegetarian approach.
These supplements can be taken before eating, in the middle of the meal, or at the end of the meal.

SUPPLEMENTS DO NOT NEED TO BE TAKEN WITH UNRESTRICTED MEALS.
1 to 3 (specify__________) UNRESTRICTED MEALS ARE ALLOWED EACH WEEK.
In an unrestricted meal there are no restrictions on the quantity of animal-derived protein (meat, fish, cheese, eggs) that can be eaten.

FURTHER ADVICE FOR PREGNANT WOMEN
DRESSINGS / COOKING METHODS

All foods can be cooked as preferred: stewed, steamed, grilled, broiled, baked, or occasionally fried (in extra virgin olive oil or peanut oil).
It is preferable to use extra virgin olive oil for seasoning and cooking, avoiding the use of mixed seed oil, butter, lard, margarine, cream, sauces (such as mayonnaise, ketchup, tuna sauce, etc.), and items containing sodium glutamate.
You should avoid using vegetable fats, non-hydrogenated vegetable fats, palm oil and coconut oil.
It is preferable to use bakery products (crackers, bread sticks) without added fat or those made with extra virgin olive oil, sunflower or corn oil.
Spices and herbs (such as rosemary, sage, basil, oregano, thyme, parsley), chilli peppers, onion, garlic, lemon juice, vinegar, balsamic vinegar, miso, tamari, shoyu, etc . can be used as desired.
To increase the intake of minerals (calcium, iron, potassium, etc.) sesame seeds, sunflower seeds or pumpkin seeds can be used (up to 1 tablespoon per day).

TOXOPLASMOSIS

If you are receptive to toxoplasmosis, the meat you eat during unrestricted meals should always be well-cooked.
Eat only cooked vegetables; it should be noted that freezing eliminates toxoplasma.
Caution should also be used in preparing strawberries and other berries, mushrooms and herbs (parsley, basil, sage, etc.).

**LISTERIOSIS**<br>To prevent listeriosis, the following products must be avoided: vacuum-packed products (such as smoked salmon, sliced cheese, etc.), unpasteurized milk and cheese made with unpasteurized milk.<br>**DRINKS**<br>We recommended that you drink water (still or sparkling) throughout the day. Consult your nephrologist to find out how much water you should drink each day.<br>Note that in pregnancy wine and beer should be drunk only occasionally and in small quantities. Cocktails, spirits and liqueurs are forbidden.<br>Due to their high sugar content, avoid soft drinks, syrups, bottled fruit juices and soluble herbal teas.<br>**SWEETS**<br>Reduce your consumption of foods with a high sugar content: white and brown sugar, ice cream, honey, malt, fruit jellies, pastries,, cakes, puddings, chocolate, cookies, candy, etc..<br>You should not use artificial sweeteners like aspartame (E951), acesulfame K (E950), saccharin (E954) and sucralose (E955), cyclamate (E952) or neohesperidin DC (E959).<br>Finally, keep a food journal for three or four days before each of your clinical visits, as a basis for discussion with your nephrologist or dietician.<br><br>***To make your life simpler***<br>*You can eat everything that grows in the soil, under the soil and on trees, and everything derived from what grows in and under the soil and on the trees. The amount you eat is only limited by weight gain: be careful about how much fruit you eat, as fruit contains sugars, and only limited amounts of sugar are allowed in pregnancy. Since olives grow on trees, olive oil can be used freely, as long your weight gain does not exceed prescribed limits.*<br>*Except for the unrestricted meals, eating the flesh of any animal (mammal, bird, fish, seafood) is forbidden, as are products derived from animals (eggs and dairy products , with the exception of butter, which should only be used in limited amounts because of its high fat content).*<br>*During the unrestricted meals, you can forget about diet restrictions (but not about your weight).*	
**BREAKFAST**	At breakfast you can have tea or coffee with a small amount of milk, soy drink enriched with calcium or soy yogurt; cookies; rusks or bread with jam; cereal (such as corn flakes, oatmeal or muesli), or a slice of homemade cake (butter, oil, and a small amount of milk or yogurt are allowed).<br>Alternatively you can have bread with olive oil and tomatoes or olives, bread and tofu, crackers or bread sticks made with extra virgin olive oil.
**LUNCH and DINNER**	At lunch and dinner it is important to eat:<br>- a portion of pasta, rice, couscous, or cereals (like barley, millet, kamut, wheat) seasoned with vegetable oil, tomatoes, herbs or garlic. Potatoes can be eaten instead of bread or pasta.<br>- a portion of legumes (for example, chickpeas, peas, beans or lentils). Legumes should be eaten for at least one main meal (lunch or dinner) in association with pasta, rice or cereals. Other plant-based protein sources, such as tofu, tempeh and seitan can be eaten instead of legumes.<br>- vegetables of any kind, raw or cooked, (prepared in accordance with the indications for toxoplasmosis).<br>- a portion of bread or bread sticks or crackers made with extra virgin olive oil.<br>- a portion of fresh fruit (150-200 g).
**SNACKS**	Mid-morning and mid-afternoon snacks can include:<br>- 1 cup of calcium-enriched soy yogurt or soy drink<br>- bread, crackers or bread sticks made with extra virgin olive oil<br>- bread with tofu, olives or olive paste, tomatoes or other vegetables<br>- 1 serving of fresh fruit<br>- 4 to 6 oily nuts such as walnuts a day, as they have a high content of omega 3 ("good" fatty acid)..

	- raw vegetables like fennel, celery, peppers, cucumbers, tomatoes and carrots (see indications for toxoplasmosis).<br>*Since in pregnancy fructose and glucose should be limited, unless otherwise indicated, no more than 2 servings of fruit are allowed per day.*

Figure S1: *eGFR and proteinuria at referral and delivery. First point, referral; second point, delivery*.

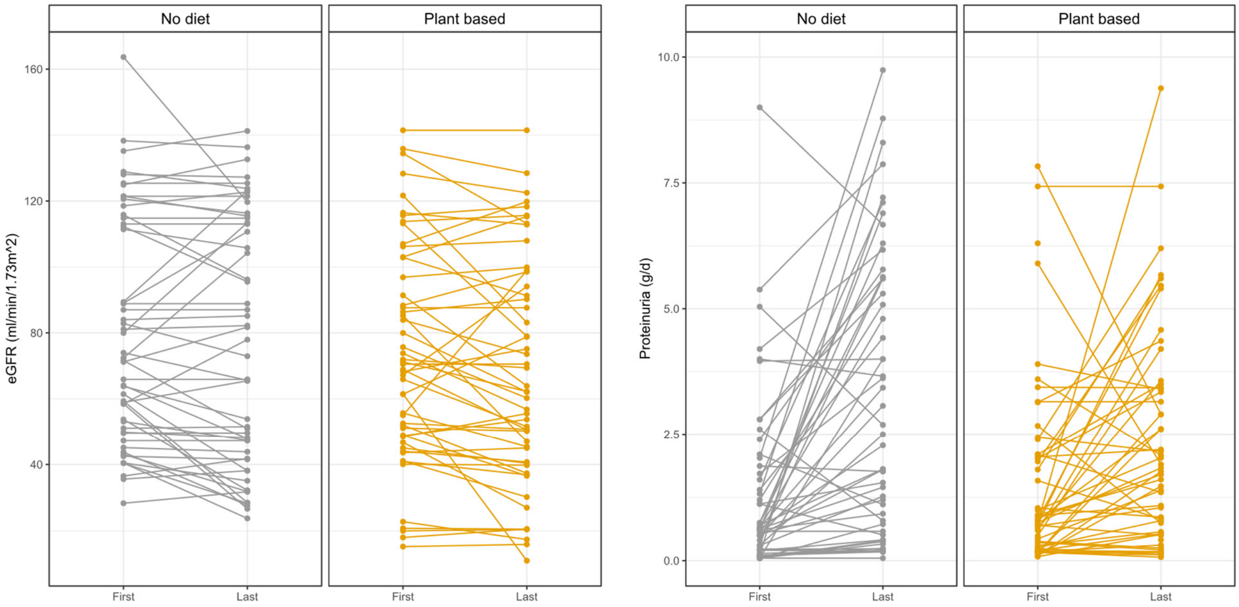

Supplement: Supplementary file 1 [file nutrients-14-04203-s001.zip › nutrients-1862239-supplementary.pdf]
